# Arginine-iron–hexametaphosphate complex as a novel nitrogen plant nutrition reducing nitrate leaching in Scots pine (*Pinus sylvestris*) seedling production

**DOI:** 10.1038/s41598-025-15665-7

**Published:** 2025-08-13

**Authors:** Tinkara Bizjak-Johansson, Marjan Bozaghian Bäckman, Lina Nilsson, Mattias Holmlund, Nils Skoglund, Torgny Näsholm, Regina Gratz

**Affiliations:** 1 Umeå Plant Science Centre (UPSC), Forest Genetics and Plant Physiology, Swedish University of Agricultural Sciences, Umeå, Sweden; 2 Thermochemical Energy Conversion Laboratory, Department of Applied Physics and Electronics, Umeå University, Umeå, Sweden; 3 Arevo AB, Dåva Energiväg 8, Umeå, Sweden; 4 Forest Ecology and Management, Swedish University of Agricultural Sciences, Umeå, Sweden

**Keywords:** Arginine–iron–hexametaphosphate, Fertilization, Nitrate leaching, Organic nitrogen, Plant nutrition, Scots pine, Plant development, Plant physiology

## Abstract

The industrial production of conifer seedlings in nurseries uses large amounts of fertilizers to ensure their proper growth and accurate nutrient status. However, inorganic nitrogen fertilization leads to nitrate leaching, which has negative environmental consequences. An alternative solution could be the use of controlled-release fertilizers that supply nutrients over longer periods and hence have a lower environmental impact. This study analysed the performance of a novel arginine–iron–hexametaphosphate complex on Scots pine (*Pinus sylvestris*) seedlings. The complex was characterized using a wide range of analytical tools, indicating that it is a precipitated complex rather than a crystalline compound. Plant growth on arginine–iron–hexametaphosphate was comparable to a commercial inorganic nitrogen controlled-release fertilizer but with significantly lower nitrate leaching. A nitrogen budget of seedlings and growth substrate showed that seedlings had acquired nitrogen in excess of the amount of nitrogen present at the start of the experiment, and this excess nitrogen was smaller in seedlings grown on the inorganic fertilizer. Measurements of acetylene reduction in seedlings indicated low but measurable rates of nitrogen fixation, potentially contributing to the excess nitrogen. Together, the results showed that the arginine–iron–hexametaphosphate complex is a good alternative to commonly used fertilizers and can contribute to sustainable seedling production.

**Supplementary Information:**

The online version contains supplementary material available at 10.1038/s41598-025-15665-7.

## Introduction

The nutrient most commonly limiting both plant and tree growth is nitrogen^1,2^ and it has, therefore, become a common practice to utilise fertilization with inorganic nitrogen to promote plant growth in both agriculture and forestry^3,4^. However, it has become increasingly clear that inorganic nitrogen fertilization can lead to severe negative consequences for soil, water and air, such as water pollution and eutrophication, acid rain, greenhouse gas emissions and biodiversity loss^5–8^. Many of these negative effects can be linked to nitrate leaching after fertilizer application^8,9^. Therefore, more sustainable alternatives for promoting plant growth are needed. One of the options is the use of organic nitrogen plant nutrition, specifically amino acid-based nutrition^10,11^. It has been shown that all studied plants are capable of taking up and are able to metabolise various amino acids^12–15^. Additionally, several amino acid transporters have been identified in plants providing further evidence of plant amino acid uptake^13,16–21^. It has been shown in several studies that, in general, organic nitrogen can promote plant growth^22–25^ while leading to lower nitrate leaching^26,27^.

In the Fennoscandian boreal forest Scots pine (*Pinus sylvestris*) is one of the main tree species and represents 45% and 27% of all planted seedlings in Sweden and Finland, respectively^28^. Pine seedlings growing in nurseries are heavily fertilized to promote their growth and ensure better survival and establishment after planting^29–31^. Actually, the amount of nutrients supplied is on average higher per tree nursery area than per agricultural land area^32^. During the production of pine seedlings, there is a high risk of nitrate leaching from the growth media because of high nitrate mobility and low root nitrate uptake^27^. Furthermore, soil overirrigation to promote seed germination and empty spaces around the seedling cassettes, as fertilizer is often applied by mobile boom systems or sprinklers, can contribute to additional nitrate leaching^32^. The leached nitrate can have a detrimental effect on the groundwater^32^. Previous studies showed that Scots pine seedlings grow well when supplemented with organic nitrogen in the nursery^23,33,34^. One of the commonly used amino acids used in organic nitrogen nutrition is arginine. Arginine is one of the main nitrogen storage forms in conifer seeds, bark and needles^35–37^ and it has been shown that pine can absorb substantial amounts of intact arginine with higher uptake rates compared to nitrate and similar or higher compared to ammonium^38–40^. Furthermore, arginine has been chosen for plant nutrition as it is the most nitrogen-rich amino acid and is positively charged in the pH range of the growth substrate, leading to higher retention in the soil and lower leaching^27,41^. Additionally, the assimilation of organic nitrogen into proteins has a lower carbon cost compared to inorganic nitrogen, which results in a carbon bonus often leading to a higher growth rate and nitrogen use efficiency by the plants^25,40,42^. Several studies showed that fertilization with arginine can lead to a higher root-to-shoot ratio^23^ and higher mycorrhization of conifer roots compared to inorganic nitrogen fertilizers^23^, both beneficial for seedling establishment in the field as it depends on the ability to acquire enough water^43,44^.

Arginine-based liquid plant nutrition has been used for cultivating conifer seedlings in nurseries for several years^23,45^. A more recent discovery pointed to the possibility of formulating arginine-based controlled-release plant nutrition by reacting arginine with various phosphate compounds. To that end, crystals of arginine monophosphate have been shown to release nutrients over extended periods of time, providing nitrogen and phosphorus for planted seedlings^46–49^.

Controlled-release fertilizers are usually mixed into the growth media when planting to provide the plants with nutrients over months based on the plant’s needs, which contributes to lower leaching^50,51^. But most of these fertilizers are encased in largely petroleum-based synthetic polymer coats, which are fossil-fuel derived and non-biodegradable^51^. It has been proposed that controlled-release and biodegradable arginine-based organic nutrition could be produced by complexing an amino acid to polyphosphate and precipitating the complex with the addition of metal ions (e.g. iron, calcium, manganese, etc.) to produce a solid phase complex^52^. By adjusting the length of the polyphosphate, the nitrogen availability could be shortened or prolonged, changing the amount provided to the plant at a specific time^52^.

The main aim of this study was to investigate the chemical properties of arginine-based controlled-release nutrition under development and test if it can promote Scots pine seedling growth in a greenhouse setting similarly to commercially available inorganic nitrogen controlled-release fertilizer (referred to as commercial CRF). An explorative chemical analysis was executed to characterise the chemical properties of the novel complex arginine–iron–hexametaphosphate (referred to as Arginine Fe-HMP), produced by Arevo AB from arginine, hexametaphosphate and iron. To test the efficacy of this complex, a six-month-long greenhouse experiment was performed to analyse the seedling growth promotion. The hypotheses addressed in this study were: (a) Treatment with Arginine Fe-HMP will lead to lower nitrate leaching compared to commercial CRF and (b) application of Arginine Fe-HMP will promote Scots pine seedling biomass comparable to commercial CRF.

## Materials and methods

### Arginine Fe-HMP synthesis

Dry powders of 3.6 moles of L-arginine free base (Millipore) and 0.4 moles of hexasodium hexametaphosphate (Sigma-Aldrich) were thoroughly mixed. The mixture was then dissolved in 7.2 L of 20 °C tap water in a closed 10-l container under stirring. When the L-arginine free base and hexasodium hexametaphosphate were fully dissolved, 1.8 moles of ferrous sulphate heptahydrate (Sigma-Aldrich) were slowly added to the solution. The resulting mixture was stirred for 1 h. The mixture was allowed to stand for 14 h, and the resulting precipitate was extracted with a Büchner filtration set-up. The precipitate was dried at 55 °C for 5 days.

### Chemical analysis

#### Elemental analysis

Arginine Fe-HMP was sent for element analysis to an accredited laboratory, where the element content was measured for carbon, nitrogen, aluminium, boron, calcium, cadmium, copper, iron, potassium, magnesium, manganese, molybdenum, sodium, nickel, phosphorus, sulphur, silicon and zinc. The content of carbon and nitrogen was analysed using the Dumas method^53^, while the content of other elements was analysed using inductively coupled plasma optical emission spectroscopy (ICP-OES)^54^.

#### Scanning electron microscopy with energy-dispersive X-ray spectroscopy (SEM-EDS)

SEM-EDS was used to analyse the Arginine Fe-HMP elemental composition and morphology. Arginine Fe-HMP was first milled into a fine powder using a mortar and pestle. The finely milled material was then mounted onto double-sided carbon tape before being inserted into the instrument chamber for analysis. Scanning electron microscopy (SEM) and energy-dispersive X-ray spectroscopy (EDS) were carried out using a Carl Zeiss EVO LS15 microscope equipped with an Oxford Instruments X-Max 80 mm^2^ detector. SEM imaging and EDS elemental analysis were performed with backscattered electrons at an accelerating voltage of 15 kV and a probe current of 700 pA. To determine the average elemental composition, four area analyses (1.5 × 1.5 mm^2^) were conducted, complemented by spot analyses to assess local phase variations and compositional differences.

#### Fourier-transformed infrared spectroscopy (FTIR)

FTIR spectroscopic analysis was performed to analyse the functional groups present in Arginine Fe-HMP. The analysis was done at the Vibrational Spectroscopy Core Facility at Umeå University, using a Bruker IFS 66v/S vacuum bench spectrometer (Bruker Optik GmbH). To prevent total absorbance, Arginine Fe-HMP was ground using a mortar and pestle with KBr powder (IR transparent agent) in a 1:5 ratio until a homogenous mixture was achieved. Spectra from pure KBr powder as background and Arginine Fe-HMP were collected over the spectral range of 4000–400 cm^− 1^ at 4 cm^− 1^ spectral resolution.

Spectra standardization was performed in OPUS (Version 7, Bruker Optik GmbH), using the built-in functions. First, baseline correction was performed using a 64-point rubber band. Then, vector (total area) normalization was performed over the entire spectral region followed by an offset correction in the 3800–4000 cm^− 1^ region.

#### Powder X-ray diffraction (XRD)

XRD analysis was conducted to identify whether Arginine Fe-HMP contained original reactants or if the material contained other known structures. Arginine Fe-HMP was ground using a mortar and pestle. The measurements were performed using a Bruker AXS D8 Advance powder diffractometer using a Lynxeye XE-T detector with Cu Kα radiation and a 2θ scanning range of 10–70°. The data was collected as a θ–θ scan with Cu Kα-radiation generated at 40 kV and 40 mA in the 2θ range of 10–70° with a sample rotation of 5° per minute. Phase identification was carried out in DIFFRAC.EVA 7.2 (Bruker AXS GmbH, Karlsruhe, Germany, 2024) using the Powder Diffraction File (PDF) 5 + 2024 database^55^. The PDF numbers next to chemical compounds refer to the chemical compound identification number in the PDF-5 + 2024 database.

#### Synchrotron-based X-ray absorption spectroscopy (XAS)

Balder beamline at the synchrotron MAX IV Laboratory, Lund, Sweden was used to conduct XAS at the Fe K-edge utilizing XANES for investigation of oxidation state and EXAFS to explore if Fe atoms display long-range order in the precipitated complex. Samples were finely milled using a pestle and mortar and mixed with polyethylene before pellets were pressed with a diameter of 13 mm, which was made to ensure a suitable optical density for absorption analysis in transmission mode. The beamline was equipped with a Si[111] double crystal monochromator and the data were collected in transmission mode with 5 scans. Energy calibration was performed using iron foil with E0 set to the Fe K-edge at 7 112 eV.

### Greenhouse experiment

#### Set-up, run-off water, germination and survival

The greenhouse experimental research on plants, including the collection of plant material, complied with all relevant institutional, national, and international guidelines and legislation. The experiment included three treatments: control, novel plant nutrition Arginine Fe-HMP and commonly used commercial CRF Osmocote (Substral). Each treatment included three cassettes with 60 planted seeds each, which were protected from the edge effect by being surrounded with extra Scots pine seedling cassettes. The cultivation cassettes used are standard, hard-plastic cultivation cassettes used in Scandinavia, Starpot50. One cassette holds space for 60 seedlings, with a respective substrate volume of 50 ml. The exact measures are: height 90 mm, upper inner diameter per each cultivation spot 35 mm and lower inner diameter 16 mm. The growth media used was non-fertilized, limed peat (referred to as soil throughout the manuscript) and the same amount of peat was used for all treatments. The nutrition (Arginine Fe-HMP and commercial CRF) was mixed into the soil before it was distributed in the cassettes. The amount of added nutrition was calculated so that each seedling received 12 mg of nitrogen to equalise the amount of nitrogen added to the soil for both nitrogen treatments. The cultivation of seedlings in nurseries extends over several months and controlled-release fertilizers are typically used in the early phase of the cultivation cycle. The choice of 12 mg N was based on an estimate of N required for growing seedlings through this early phase. The control cassettes did not receive any added nitrogen. Besides the one-time application of either Arginine Fe-HMP (Table 1) or Osmocote at a rate of 12 mg N, no further products were applied in any of the cassettes. Scots pine seeds (from the seed orchard Domsjöänget 411 and donated from the forest company Holmen AB) were placed in the cassettes and covered with sawdust.

**Table 1:** Elemental analysis of the arginine Fe-HMP complex composition using Dumas method and ICP-OES.

Element	Molar amount (mmol kg^− 1^)
Carbon	17,318
Nitrogen	11,566
Phosphorus	1927
Iron	1254
Sodium	561
Sulphur	312
Calcium	5.34
Potassium	4.86
Manganese	3.28
Magnesium	2.96
Boron	2.68
Silicon	1.14
Aluminium	0.67
Zinc	0.14
Nickel	0.09
Copper	< 0.08
Molybdenum	< 0.02
Cadmium	< 0.01

To analyse the leaching of several nutrients, each cassette with the plants was watered with exactly 1.2 L of water after planting and before the run-off water collection on the second day. The run-off water samples were sent for nutrient analysis to an accredited laboratory. As NPK are central elements in every fertilizer, nitrogen, phosphate as well as potassium were included in the analysis. We mainly focused on nitrate as nitrogen form in the runoff analysis, based on studies of N fertilization in conifer nurseries that have shown that nitrate-N dominates in the run-off during cultivation^27,56^. Since the main components in our novel plant nutrition—in addition to nitrogen and phosphate—are iron, sodium and sulphur (see Table 1), we included those elements in the analysis. Calcium as well as magnesium we additionally included as they play essential roles for plant growth and soil structure. Nitrate was analysed using flow analysis with spectrometric detection, while the other elements were analysed using ICP-OES. The seedlings were grown in the greenhouse under a 16-h daylight and 8-hour nighttime regime, utilising FL300 sunlight LED lamps with 80% intensity. The temperature was kept at 20 °C during the day and 15 °C during the night, and the plants were watered one to two times per day depending on their water demand. The greenhouse experiment was started in November 2022 and finished in May 2023, when the seedlings were 6 months old. Every day of the first month the germination rates were recorded. Additionally, seedling survival was measured each month, and selected seedlings and cassettes were photographed monthly until the seedlings reached six months of growth.

#### Seedling biomass

After 6 months of growth, the seedling biomass (shoot length, dry root weight and dry shoot weight) was measured for 30 randomly selected seedlings per treatment. The seedlings were cut just above the first root to separate the root and shoot part of the seedling, and the shoot length was measured. The roots and shoots were oven-dried at 60 °C for at least 48 h before their dry weight was recorded.

#### Carbon, nitrogen and isotope ^15^N abundance

The carbon, nitrogen and isotope ^15^N abundance of the samples were analysed for whole seedlings, soil, seed and nutrition samples. For seedling analysis, nine seedlings per treatment were randomly selected from the seedlings used for biomass measurements and their root and shoot tissues were combined into one sample. For soil samples a composite sample was collected for each cassette. For both seeds and nutrition samples three replicates were used. All samples were oven-dried at 60 °C for at least 48 h and ground using a bead mill for 60 s with an oscillation frequency of 30 Hz. The samples were analysed using an Isotope Ratio Mass spectrometer (DeltaV, Thermo Fisher Scientific) coupled with an Elemental analyser (Flash EA 2000, Thermo Fisher Scientific) following the method by Werner, et al.^57^.

#### Chlorophyll content

Chlorophyll content was analysed based on Warren^58^ and Arnon^59^ with 12 randomly selected seedlings per treatment. Shortly, all seedling needles were collected and cut into smaller pieces. The needles were placed into a tube and 7 ml of DMSO was added before samples were incubated at 60 °C for 6 h. After incubation, DMSO was added to the final sample volume of 10 ml. The absorbance of 200 µl of the supernatant was measured at 645 nm and 663 nm using a plate reader (BioTek Epoch), where three technical replicates were used for each biological replicate. The total chlorophyll content was calculated based on the equation presented in Arnon^59^ and the pathlength correction was calculated based on Warren^58^. The supernatant was removed, and the samples were dried at 60 °C for at least 48 h before their dry weight was measured. The dried weight was used to normalise the chlorophyll content values.

#### Ergosterol measurement

Roots of 15 seedlings per treatment were collected, freeze-dried and ground using a bead mill. The extraction protocol used in this study was based on a scaled-down version of the original protocol by Salmanowicz and Nylund^60^ which was published by Martin^61^ for free ergosterol extraction from ectomycorrhizal roots. One ml chloroform/methanol (50/50, v/v), containing 1.125 ng µl^− 1^ ergosterol-D3 as an internal standard was added to a tube with 5–6 mg root material. The samples were wrapped in aluminium foil, vortexed for 30 min, and subsequently centrifuged at 13,300 g for 2 min. A 200 µl aliquot was transferred to a 350 µl vial and dried by vacuum centrifugation (Genevac miVac Quattro, Thermo Fisher Scientific). 15 µl pyridine was added to the vial and 30 µl of a mixture containing (50/50, v/v) *N*-Methyl-*N*-trimethylsilytrifluoroacetamide (MSTFA) with 1% trimethylchlorosilane (TMCS) and heptane with 15 ng µl^− 1^ methyl stearate was added. The vial was vortexed and incubated for 1 h before analysis. One µl was injected splitless by an autosampler (Agilent 7693, Agilent technologies) into a gas chromatograph (Agilent 7890 A, Agilent technologies). Separation was performed on a 10 m × 0.18 mm fused silica capillary column with a chemically bonded 0.18 μm DB 5-MS stationary phase (J & W Scientific). Detection was achieved using a triple-quadrupole mass spectrometer (Agilent 7000, Agilent technologies). Multiple reaction monitoring transitions 468 > 363 and 471 > 366.4 were used as quantitative transitions for ergosterol and ergosterol-D3 respectively. Transitions 468 > 378 and 471 > 381.2 were used as qualitative transitions for ergosterol and ergosterol-D3.

#### Acetylene-reduction assay

Acetylene-reduction assay was adapted after Bizjak, et al.^62^ to indirectly measure seedling nitrogen fixation. Namely, 10 seedlings from each treatment were randomly collected and their roots were washed with water. The seedlings were placed in sterile glass tubes, five ml of sterile water was added, and the tubes were closed with a rubber septum. For each seedling, 10% of the air was replaced with acetylene gas. The samples were incubated for two hours under constant light conditions at room temperature. After incubation, the production of ethylene was measured with GC (Shimadzu GC-8 A) coupled with a flame ionization detector. The seedlings were dried at 60 °C for at least 48 h before their dry weight was recorded. Calculated seedling ethylene production was adjusted for any ethylene present due to injected acetylene gas (water controls) and endogenous ethylene production (seedling samples without added acetylene gas) and further normalised based on the measured seedling dry weight.

### Calculations

For the nitrogen budget, calculations of nitrogen content were done per seedling. In the case of the seedling nitrogen content, the nitrogen concentration was multiplied by the exact seedling weight (*n* = 9). For soil, the nitrogen concentration was multiplied by the average dry soil weight per seedling (*n* = 3). For initial nitrogen introduced in the system, we took into account the nitrogen added through initial soil (nitrogen concentration in the soil before the experiment start multiplied by average dry soil weight per seedling, *n* = 3), seed (seed nitrogen concentration multiplied by average dry seed weight, *n* = 3) and nutrition (12 mg of N per seedling for both nitrogen treatments and 0 mg of N for control treatment). The percentage of excess nitrogen detected in the system was calculated by considering the amount of nitrogen at the end of the experiment compared to the amount of nitrogen introduced into the system at the beginning of the experiment. The nutrition use efficiency was calculated by dividing the seedling nitrogen content by the nutrition nitrogen added per seedling.

The ergosterol concentration is the amount of ergosterol per unit weight of dried root material. The ergosterol content was calculated by multiplying the ergosterol concentration with the average seedling dry root weight.

### Statistics

The statistical analysis of all data was performed using the program SPSS Statistics versions 27 and 29 (IBM). Normally distributed data: run-off water (*n* = 3), germination rate (*n* = 3), survival (*n* = 3), shoot length (*n* = 30), root-to-shoot ratio (*n* = 30), chlorophyll content (*n* = 12), dry shoot weight (*n* = 30) and dry root weight (*n* = 30), total dry weight (*n* = 30), seedling nitrogen content (*n* = 9), soil nitrogen content (*n* = 3) and both ergosterol measurements (*n* = 15), were analysed using one-way ANOVA followed by Tukey’s honest significance test (HSD) test with nitrogen treatment as a variable. Non-normally distributed acetylene-reduction assay results (*n* = 10) were analysed using the Kruskal-Wallis test followed by pairwise comparison with Bonferroni’s adjusted value.

## Results

### Arginine Fe-HMP structural characteristics

**Fig. 1:**
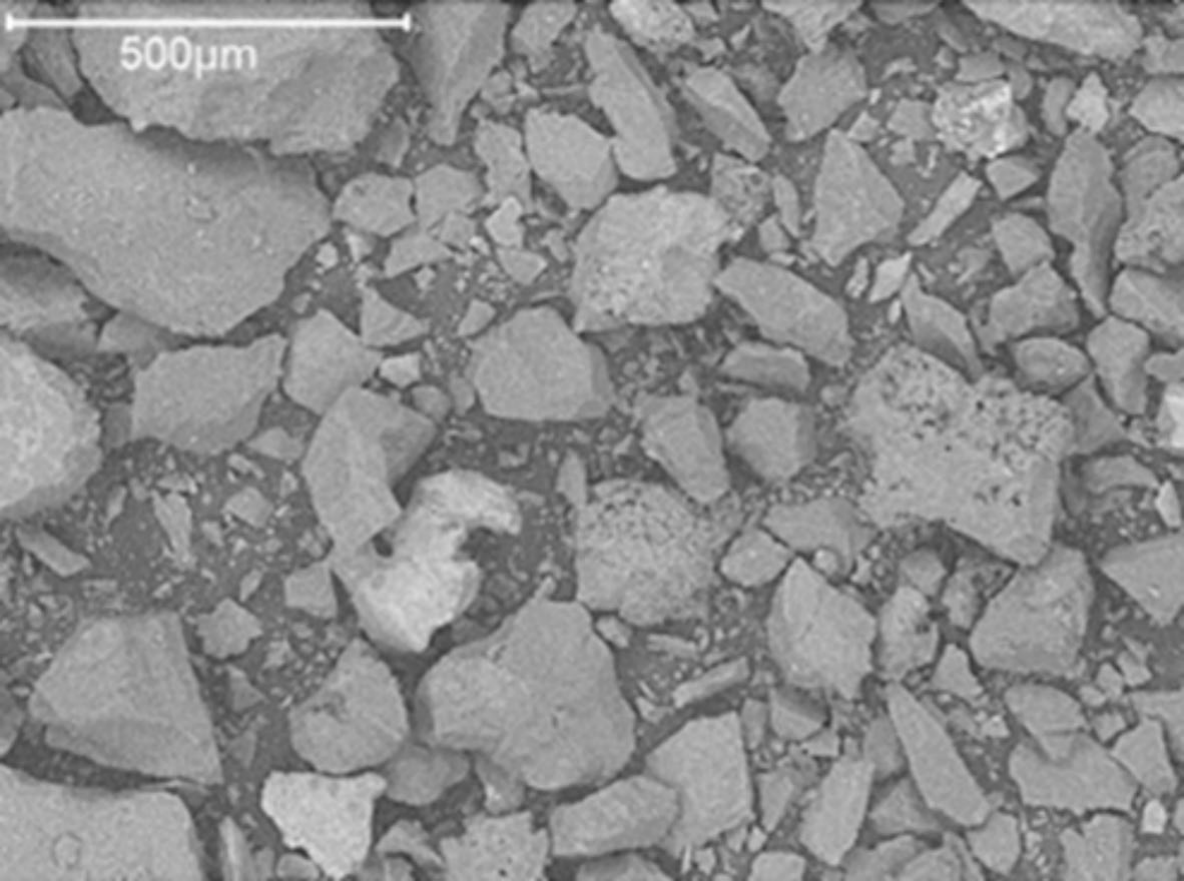
Representative image of grains in Arginine Fe-HMP based on SEM-EDS.

Several chemical analysis methods were used to investigate the structural characteristics of novel plant nutrition under development called Arginine Fe-HMP. The results from the Dumas method and ICP-OES confirmed that the composition was dominated by carbon, nitrogen, phosphorus, iron, sodium and sulphur, while the amounts of other elements were below 1% (Table 1). Similar results were obtained by SEM-EDS, which provided elemental composition analysed on a C- and O-free basis, with comparable and significant quantities of phosphorus and iron (18.5 and 17.4 atomic %, respectively), and smaller but notably similar proportions of sodium and sulphur (4.0 and 3.0 atomic %, respectively). The ratio of nitrogen-to-carbon in the analysis was 0.67, i.e. identical to the theoretical ratio of these elements in arginine, indicating that the amino acid remained intact during synthesis. The elemental ratios of other elements in the complex cannot be used to discern the exact composition of the precipitated complex due to its multi-component composition. Based on the overall ratio of carbon-to-nitrogen-to-phosphorus molar concentrations, we arrive at an arginine-to-phosphorus ratio of 1.5. The phosphate can be present as metaphosphate (PO_3_^−^), pyrophosphate (P_2_O_7_^4−^), orthophosphate (PO_4_^3−^) or a combination of these forms with various degrees of protonation. However, the Arginine Fe-HMP complex exhibits a uniform morphology according to SEM-EDS with no significant variations, suggesting a homogeneous synthesis process (Fig. 1).

**Fig. 2:**
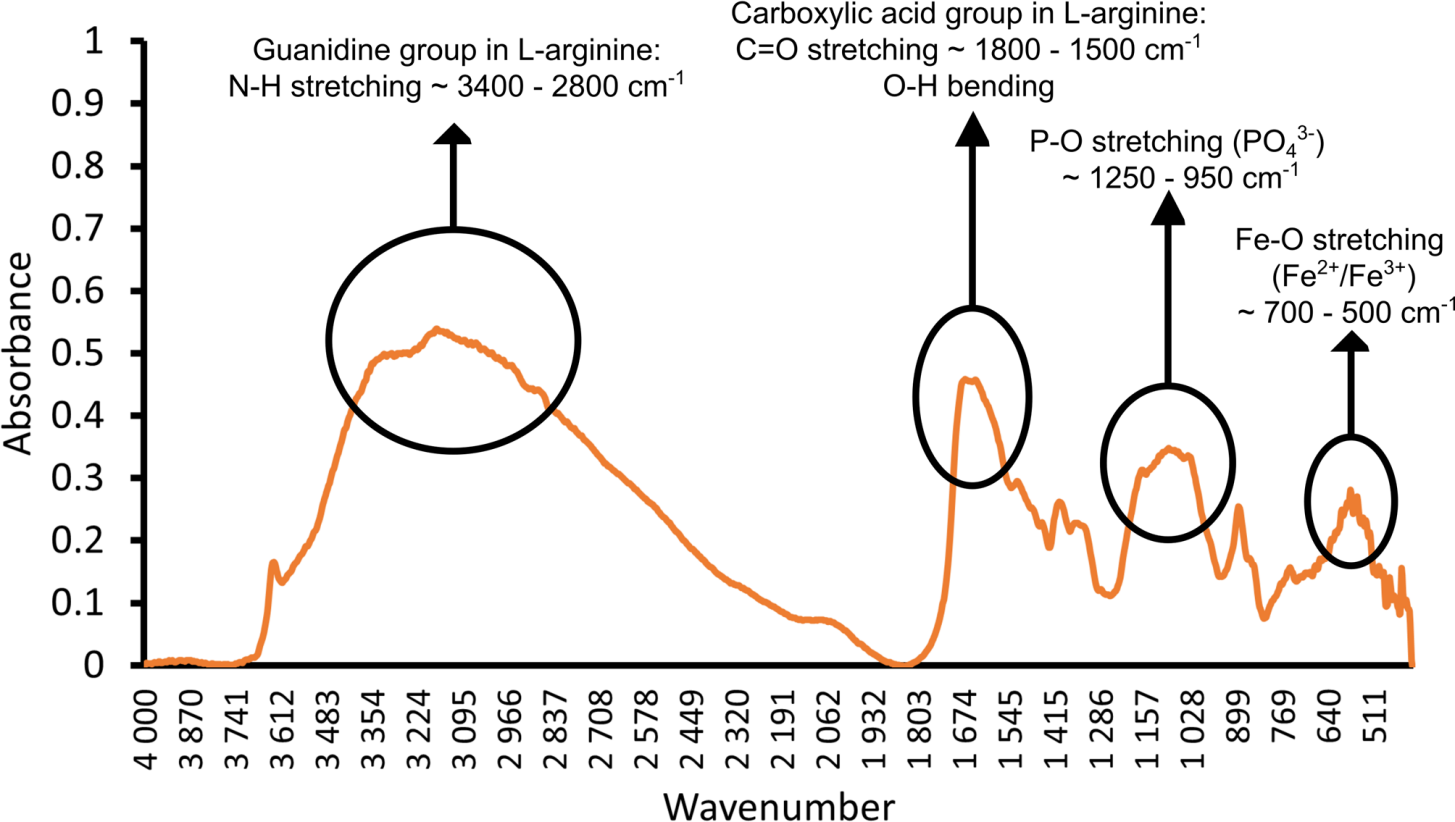
FTIR spectrum for Arginine Fe–HMP with its characteristic bands of N–H stretching (~ 3400 –2800 cm^− 1^), C= O stretching (~ 1800 –1500 cm^− 1^), P–O stretching (~ 1250–950 cm^− 1^), and Fe-O (~ 700 –500 cm^− 1^).

FTIR was used to further analyse the presence of functional groups in Arginine Fe-HMP. In its spectrum, four distinct groups of absorption bands were identified (Fig. 2). The broad band observed between 3400–2800 cm^− 1^ was attributed to overlapping N–H vibrations from the guanidine group (–C(NH)(NH2)) present in L-arginine. The band located between 1800 –1500 cm^− 1^ corresponding to the C=O stretching vibrations, and the observed bending O–H vibrations were both attributed to the carboxylic acid present in L-arginine. The broad absorption band in the range of 1250–950 cm^− 1^ was associated with the vibrational modes of the P–O bonds within phosphate groups, which generates multiple overlapping signals within this region. Finally, the band observed between 700–500 cm^− 1^ was likely attributed to Fe–O vibrations.

The results from powder XRD, which was used to analyse the crystallinity of Arginine Fe-HMP, showed that the complex composition was poorly crystalline with only small features barely reaching levels above the background curvature with generally broad peak profiles (Supplementary Fig. S1). No crystalline presence of the initial reactant chemicals Na_6_(PO_3_)_6_, Fe(II)SO_4_·7H_2_O, or L-arginine free base were identified in the material. The small peak features observed may contain traces of Na_2_H_2_(P_2_O_7_) (PDF 04-016-2957), Na_2_H_4_(SO_4_)_4_ (PDF 04-010-8132), Fe(PO_4_)·2H_2_O (PDF 04-014-3291), goethite (FeOOH, PDF 04-014-5919) and at least one component without a determined reference structure. However, the peak broadening of overlapping components prevents conclusive determination and quantification.

XAS was used to gain additional insight into iron in the Arginine Fe-HMP complex. The XANES region of XAS suggests a mixed oxidation state of Fe^2+^, Fe^3+^ and Fe–O features, showing that partial oxidation had occurred of the Fe(II) used in the production of Arginine Fe-HMP (Supplementary Fig. S2). EXAFS showed that iron in the precipitate is likely bonded to Fe–O in the first shell with possible further coordination at the second shell to Fe outside this bond distance, but after this, there were no clear bond distances observed.

### Seedling growth promotion by arginine Fe-HMP

**Fig. 3:**
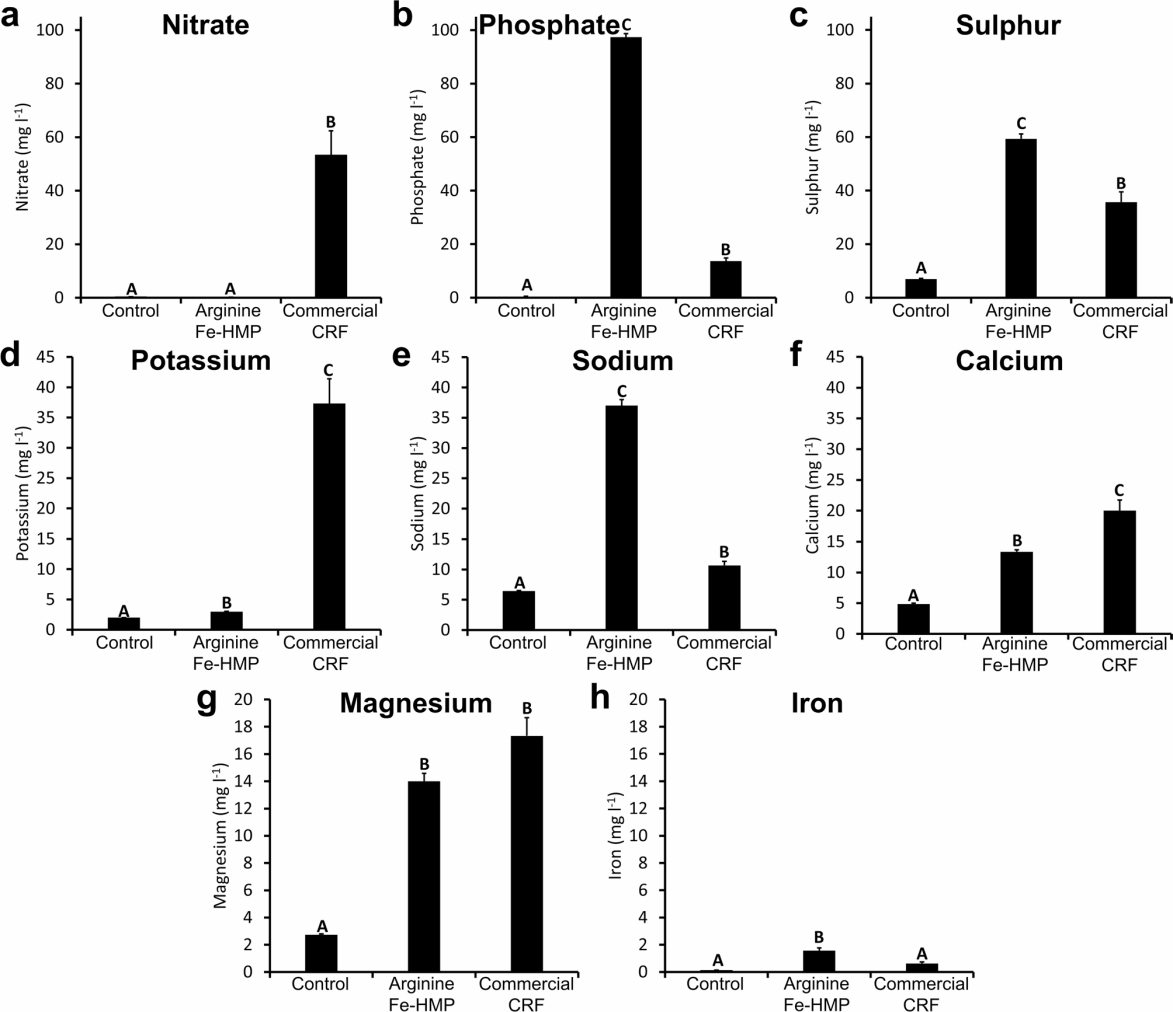
Nutrient analysis of the run-off water after planting the pines (mean + s.e.m., *n* = 3): (**a**) nitrate, (**b**) phosphate, (**c**) sulphur, (**d**) potassium, (**e**) sodium, (**f**) calcium, (**g**) magnesium and (**h**) iron using flow analysis with spectrometric detection for nitrate and ICP-OES for other elements. Different letters indicate significant differences based on ANOVA and Tukey HSD test.

A greenhouse study was used to assess Scots pine seedling growth promotion by Arginine Fe-HMP compared to control and commercial CRF treatment. The run-off water collected on the day after planting was analysed to check for leakage of essential elements (Fig. 3) such as NPK, as well the main components found in Arginine Fe-HMP, see Table 1. Commercial CRF treatment led to a significantly high nitrate leakage, while Arginine Fe-HMP and the control treatment had almost non-detectable leakage of nitrate (*p* < 0.001). Commercial CRF treatment additionally showed considerably high concentrations of potassium, magnesium and calcium in the run-off water. Arginine Fe-HMP showed high concentrations of phosphate, magnesium, iron, sulphur and sodium.

**Fig. 4:**
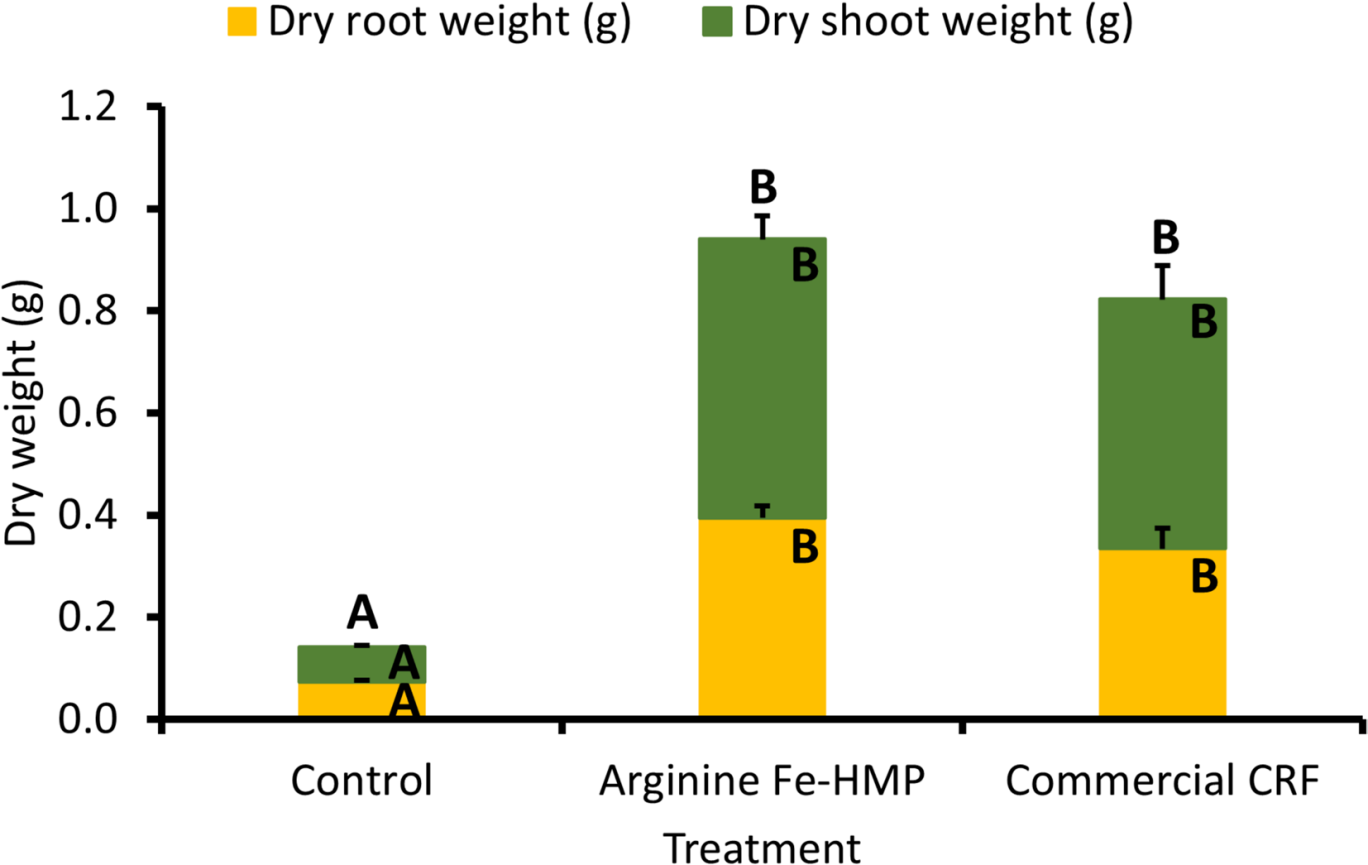
Dry shoot and root biomass measurements of the pine seedlings grown for six months (mean + s.e.m., *n* = 30) under three treatments: control, Arginine Fe-HMP and commercial CRF. Different letters indicate significant differences based on ANOVA and Tukey HSD test.

The germination rate of Scots pine seedlings was similar across all three treatments, showing no negative effect of either treatment (*p* = 0.297, Supplementary Fig. S3). After six months of growth, the survival rates of seedlings were similar, and not statistically significantly different across the three treatments (*p* = 0.655, Supplementary Fig. S4a). The shoot length of measured seedlings was significantly longer for commercial CRF and Arginine Fe-HMP compared to the control treatment (*p* < 0.001, Supplementary Fig. S4b), leading to a higher root-to-shoot ratio in control seedlings compared to the two nitrogen treatments (*p* < 0.001, Supplementary Fig. S4c). While needle chlorophyll content was similar across all treatments (*p* = 0.707, Supplementary Fig. S4d), higher variation was observed for the commercial CRF seedlings. Biomass measurements (Fig. 4) and observations (Supplementary Fig. S5) showed statistically significantly higher total dry biomass for the Arginine Fe-HMP and the commercial CRF seedlings compared to the control treatment (*p* < 0.001). This was due to both higher root and shoot dry weights for nitrogen treated compared to control seedlings (for both *p* < 0.001). The total dry weight tended to be higher for Arginine Fe-HMP compared to the commercial CRF seedlings, however, the difference was not statistically significant in part due to quite high variation seen for the commercial CRF seedlings.

**Fig. 5:**
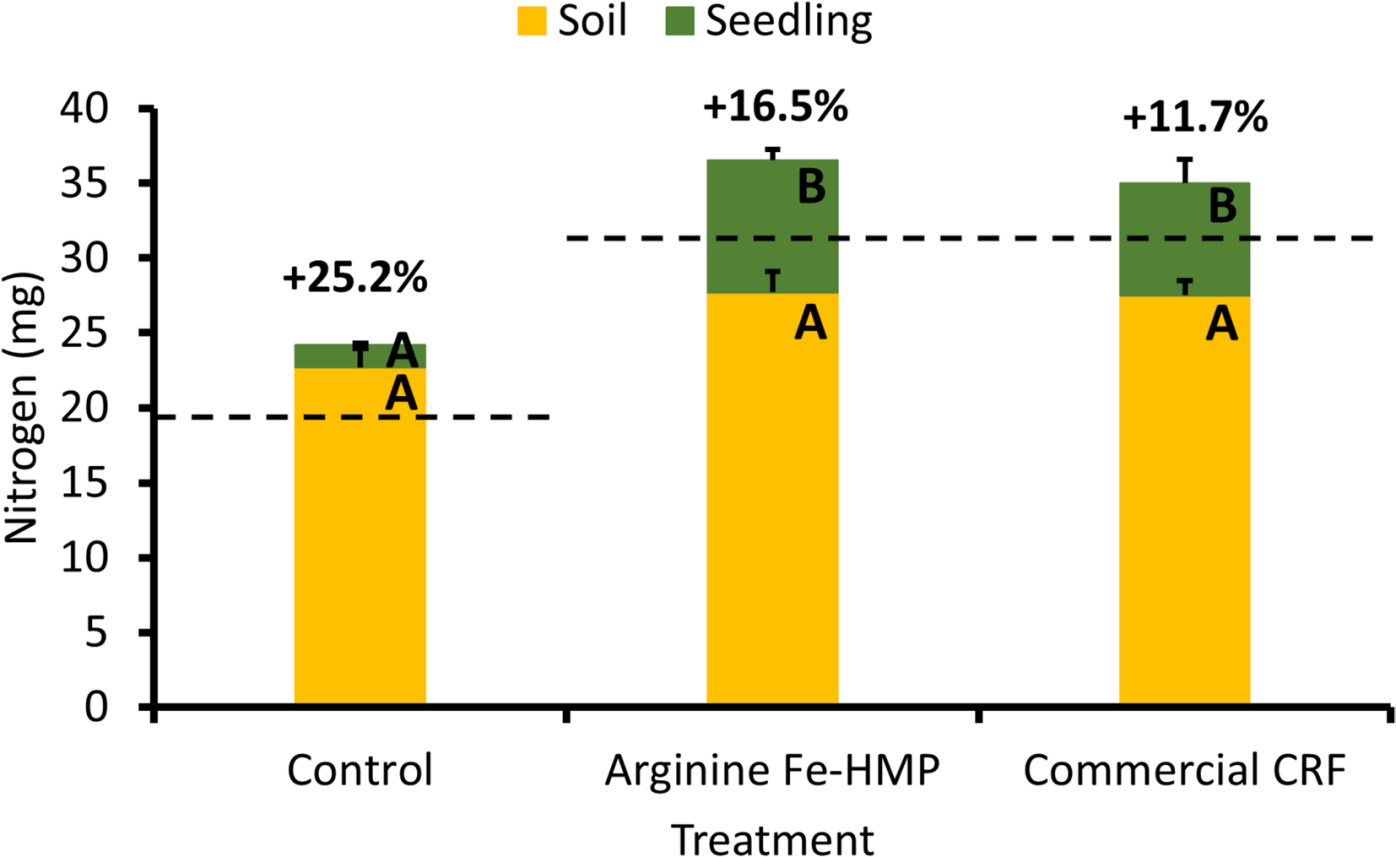
Nitrogen budget per seedling for each treatment based on measured soil (mean + s.e.m., *n* = 3) and seedling (mean + s.e.m., *n* = 9) nitrogen. The black dashed line represents the initial nitrogen introduced to the system through seed, initial soil and nutrition application (for commercial CRF and Arginine Fe-HMP treatment). The number next to the column is the percentage of excess nitrogen based on the initial nitrogen. Different letters by error bars indicate significant differences based on ANOVA and Tukey HSD test for either soil or seedling.

A nitrogen budget was constructed for each seedling, considering the nitrogen introduced into the system at the beginning of the experiment and the nitrogen detected at the end of the experiment (Fig. 5, Supplementary Fig. S6). For all treatments, there was excess nitrogen (meaning nitrogen not accounted for) detected. The fraction of excess nitrogen was 11–25%, with the commercial CRF seedlings having the lowest excess nitrogen and control seedlings having the highest nitrogen excess (Fig. 5). Further, the Arginine Fe-HMP showed a higher nutrition use efficiency of 73% compared to the commercial CRF seedlings 63% (Supplementary Fig. S6). The measurements of ^15^N isotope abundances showed distinct ∂ ^15^N values between the initial soil and seed and the soil and seedling after the 6-month-long greenhouse experiment (Table 2). Additionally, after the end of the greenhouse experiment, there were changes in the seedling and soil ∂ ^15^N values between the three treatments.

**Table 2:** Measured isotope ∂ ^15^N (‰) in initial soil (mean, *n* = 3), seed (mean, *n* = 3) and nutrition (mean, *n* = 3) together with soil (mean, *n* = 3) and seedling (mean, *n* = 9) after six months of growth for three treatments: control, arginine Fe-HMP and commercial CRF.

	Component	Control	Arginine Fe-HMP	Commercial CRF
Start of the experiment ∂ ^15^N (‰)	Initial soil	− 2.77	− 2.77	− 2.77
	Seed	3.67	3.67	3.67
	Nutrition		− 4.69	0.49
End of the experiment ∂ ^15^N (‰)	Soil	− 1.88	− 1.85	− 0.89
	Seedling	1.13	− 1.53	1.24

**Fig. 6:**
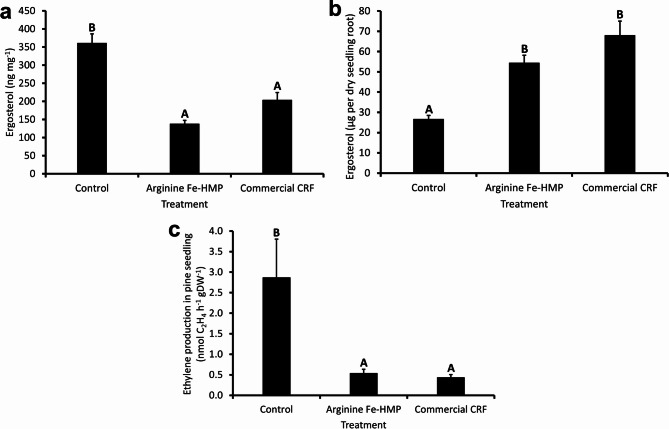
The effect of the control, Arginine Fe-HMP and commercial CRF treatment on the microorganisms: (**a**) ergosterol concentration in the freeze-dried roots (mean + s.e.m., *n* = 15, different letters indicate significant differences based on ANOVA and Tukey HSD test) and (**b**) ergosterol content per whole dry root system of each seedling (mean + s.e.m., *n* = 15, different letters indicate significant differences based on ANOVA and Tukey HSD test) and (**c**) measured ethylene production in pine seedling indicating nitrogen fixation using acetylene-reduction assay (mean + s.e.m., *n* = 10, different letters indicate significant differences based on Kruskal–Wallis pairwise comparison with Bonferroni’s adjusted value).

The effect of the nitrogen treatment on the presence and activity of microorganisms was analysed by measuring the ergosterol content in pine seedling roots and nitrogen fixation in the seedlings. The ergosterol measurements showed a significant difference in fungal abundance between the control treatment compared to both Arginine Fe-HMP and commercial CRF seedlings (*p* < 0.001), while there was no significant difference between the two nitrogen treatments. Control seedlings had the highest ergosterol concentration but the lowest total ergosterol content, while both Arginine Fe-HMP and commercial CRF displayed lower concentrations but higher contents of ergosterol (Fig. 6a,b). Nitrogen fixation, as assessed by acetylene reduction to ethylene was analysed on washed seedlings and results showed that the rate of nitrogen fixation per unit weight of dried seedling material was highest for control seedlings and lower for Arginine Fe-HMP and commercial CRF seedlings (*p* = 0.005, Fig. 6c).

## Discussion

Inorganic nitrogen-based controlled-release fertilizers are used in forestry nurseries to produce seedlings for planting, however, due to their relatively high nitrate leaching and the use of non-biodegradable synthetic polymer coats^51,63,64^ more sustainable alternatives are needed. One suggested alternative is a controlled-release organic nitrogen complex including an amino acid, phosphorus and iron; where polyphosphate length would control the nutrient release rate^52^. In this study, we synthesized and tested one such complex, Arginine Fe-HMP, analysing its composition, leaching, and ability to promote seedling growth in a greenhouse experiment.

In accordance with our first hypothesis, the chemical analysis determined the relative ratios of arginine, phosphate and iron and explored the structural characteristics of the Arginine Fe-HMP complex. Specifically, the element analysis showed a high quantity of carbon and nitrogen present in Arginine Fe-HMP (Table 1). Based on the data shown in Table 1, the ratio of nitrogen to carbon in the complex (0.668) was similar to the theoretical arginine nitrogen to carbon ratio (0.67), suggesting arginine was not decomposed during the production process. We also detected high concentrations of phosphorus and iron and somewhat lower concentrations of sodium and sulphur (Table 1), suggesting that the synthesis process effectively incorporated the target elements into the Arginine Fe-HMP complex. Additionally, the relatively high quantities of iron and phosphorus suggest their critical roles in the structural integrity or functional characteristics of Arginine Fe-HMP. A uniform elemental distribution and morphological consistency were observed for Arginine Fe-HMP (Fig. 1). Further, the FTIR findings confirmed the molecular composition and presence of functional groups in the sample (Fig. 2). The observed shifts in absorption peaks indicated interactions between L-arginine and phosphate as well as potential coordination with iron, supporting the successful synthesis of the intended molecule. The powder XRD results showed poor crystallinity in Arginine Fe-HMP, which may improve its solubility and enhance its bioavailability to plants. The results from XAS suggested iron is present bonded as Fe–O in the first shell with only small contributions in the second shell likely from Fe, pointing towards the formation of hydrated Fe ions either as complexes as [Fe(H_2_O)_6_]^+2/+3^ or Fe(OH)_2_^+^ previously mentioned in precipitation of iron and arginine by Bhattacharyya, et al.^65^. They also noted that presence of other bases—in our case phosphate—may reduce direct Fe–N interactions. The alternating layer structure present in L-arginine phosphate monohydrate^66,67^ with stacked arginine molecules with interstitial layers of orthophosphate (Supplementary Fig. S7) may have an analogue in the Arginine Fe-HMP complex. The same double-layer of arginine molecules could accommodate either hexametaphosphate rings or parts of it, depending on whether they have been hydrolyzed. Further, such monolayers of inorganic components could accommodate hydrated Fe ions in accordance with the study by Bhattacharyya, et al.^65^. To determine if such repeating structure is present in the Arginine Fe-HMP complex and attempt quantification of phosphate types, dedicated studies on the material synthesis are required to fully characterize the interactions between L-arginine, phosphate, and iron in the Arginine Fe-HMP complex with complementary analytical techniques to those employed in this study.

According to the second hypothesis, the run-off water experiment showed that control and Arginine Fe-HMP treatment had very low nitrate leaching compared to high levels observed for commercial CRF (Fig. 3). Similar results of lower nitrate leaching with organic nitrogen nutrition based on arginine were observed previously^26,27^ suggested to be due to the arginine adsorption to soil particles^27,33^. These results are important as it has been proven that nitrate leaching poses a threat to the environment and even to human health^68–70^.

The six-month-long greenhouse experiment showed that the Arginine Fe-HMP did not negatively affect either seedling germination or survival rate. In many previous studies, a higher root-to-shoot ratio was reported after organic nitrogen fertilization^23,49^, however, in our study, the Scots pine seedlings cultivated on the commercial CRF and Arginine Fe-HMP treatment had a similar root-to-shoot ratio. This could be because plants were supplied with a small amount of nitrogen merely at the beginning of the experiment, and differences might only be noticeable with higher nitrogen additions. Additionally, some previous studies have reported no differences in the root-to-shoot ratio between inorganic and organic nitrogen-treated seedlings during nursery growth^34,40^, indicating that the root-to-shoot ratio could be influenced by several environmental factors. Indeed, it has been previously reported for other plant species that soil texture and amounts of specific mineral nutrients had a significant effect on the root-to-shoot ratio^71–73^. Furthermore, even though higher chlorophyll content has been observed for conifer species under organic compared to inorganic nitrogen treatment^22,25^, we did not see any change in the chlorophyll content. Similar to the root-to-shoot ratio, it could be that differences in chlorophyll content would only be noticeable with higher nitrogen addition rates during the greenhouse experiment. However, the dry biomass results corroborated our third hypothesis that Arginine Fe-HMP was able to increase Scots pine seedlings biomass similar to commercial CRF treatment (Fig. 4). Both dry shoot and root weight as well as shoot length were comparable between the commercial CRF and Arginine Fe-HMP treatment. Similar results were previously observed for arginine nutrition, where the seedling biomass was comparable with inorganic nitrogen-based fertilization^23,34^. However, this is the first study showing similar plant growth promotion by arginine polyphosphate-based nutrition. Interestingly, the visual assessment of seedlings indicated a higher heterogeneity between the commercial CRF seedlings, while Arginine Fe-HMP seedlings were more homogenous. Similarly, a higher variation in dry biomass and chlorophyll content was observed for the commercial CRF compared to the Arginine Fe-HMP treatment. The reason for the observed higher variation of the commercial CRF could be that the commercial CRF prills are non-uniform in size, which could lead to differences in the amount of nitrogen received per seedling, even though the greenhouse experiment set-up tried to minimise these differences.

The total nitrogen budget showed the existence of excess nitrogen, which was unaccounted for after considering nitrogen amounts added through initial soil, seed and nitrogen treatment (Fig. 5). The presence of excess nitrogen indicated other nitrogen input sources such as nitrogen fixation. Scots pine does not form symbiotic relationships with nitrogen-fixing bacteria but may, under certain conditions, promote the activity of such microbes. Conditions that may promote nitrogen fixation include low levels of inorganic nitrogen compounds, and abundant supply of phosphorus and reductants such as carbohydrates^74–77^. We tested this hypothesis, employing the acetylene reduction assay, and these measurements indicated low but still measurable levels of nitrogen fixation. The measurements were, however, only performed on soil-free seedlings, and hence, it is possible that higher activities would have occurred in the soil. Looking further into^15^N abundances, soil from the control treatment at the end of the experiment displayed ∂^15^N values significantly higher than at the start of the experiment, pointing at active nitrogen fixation in this treatment^78,79^. Additionally, control seedlings displayed a high ∂ ^15^N abundance and while this in part was due to the high ∂ ^15^N abundance of the seed, the total nitrogen contribution from seed nitrogen to seedling nitrogen was low. Also, for the Arginine Fe-HMP treatment, soil and seedling ∂ ^15^N at the end of the experiment were higher than the ∂ ^15^N of soil at the beginning of the experiment and, in particular, compared to the ∂ ^15^N of the added nutrient arginine. We speculate that this difference may also result from active nitrogen fixation in this treatment, but additional studies are needed to test this hypothesis. Even for the commercial CRF treatment, a shift to higher ∂ ^15^N values at the end of the experiment was noted for soil and seedlings in comparison to soil and nutrient nitrogen at the start of the experiment. However, seedling ∂ ^15^N values higher than that of nutrition suggest nitrogen fixation was not contributing significantly to seedling nitrogen, as this contribution would have resulted in ∂ ^15^N values closer to zero. The results point to that the excess nitrogen detected in this study could be due to nitrogen fixation. It has previously been shown that conifers can receive substantial amounts of nitrogen through biological nitrogen fixation when growing in severely nitrogen-limited environments^80,81^.

Arginine Fe-HMP had a comparable effect on microbial abundance and activity to the commercial CRF (Fig. 6). Fungal abundance was similar between the two treatments, which was unexpected, as previous studies often reported a higher root mycorrhization by organic compared to inorganic nitrogen treatment^23,82^. However, some previous studies did not report any differences in fungal abundance between inorganic and organic nitrogen treatments for either nursery or planted seedlings^40,41,45^. Furthermore, similar effects of inorganic and organic nitrogen treatment were observed for seedling bacterial nitrogen fixation. As far as we know, no previous studies have analysed the effect of organic nitrogen treatment on nitrogen fixation rates in conifer seedlings. A few studies showed that organic nitrogen sources such as manure did have a positive effect on the population of nitrogen-fixing bacteria and their activity in connection to agricultural plant species^83,84^. As Arginine Fe-HMP is not a fully developed product, the results suggest that its development should target minimizing the effects on the microbiome and rather promoting the microbiome abundance and activity.

## Conclusions

Our study showed that the synthesis of a controlled-release Arginine Fe-HMP nutrition effectively incorporated the initial reagents. However, an exact chemical model of the resulting non-crystalline complex could not be determined using the range of techniques employed in this study. We found significantly lower nitrate leaching but similar Scots pine seedling growth promotion of the novel Arginine Fe-HMP nutrition compared to commercially available inorganic nitrogen controlled-release fertilizers. Thus, Arginine Fe-HMP represents a more environmentally sustainable alternative for plant growth promotion. The Arginine Fe-HMP is a novel technology still under development, hence there is a big opportunity to enhance its performance as a nutrient delivery system for plant growth even further.

## Supplementary Information

Below is the link to the electronic supplementary material.

Supplementary Material 1

## Data Availability

The data that supports the findings of this study are available from the corresponding author upon reasonable request.
