# Supplementary material for: Arginine-iron–hexametaphosphate complex as a novel nitrogen plant nutrition reducing nitrate leaching in Scots pine (Pinus sylvestris) seedling production
Source: Sci Rep. 2025 Aug 13;15:29619. doi: 10.1038/s41598-025-15665-7 (PMC12343847; doi:10.1038/s41598-025-15665-7)
Supplement: Supplementary file 1 — Supplementary Material 1 [file 41598_2025_15665_MOESM1_ESM.pdf]

# Supporting information

## Arginine-Iron–Hexametaphosphate Complex as a Novel Nitrogen Plant Nutrition Reducing Nitrate Leaching in Scots Pine (*Pinus sylvestris*) Seedling Production

Tinkara Bizjak-Johansson[1], Marjan Bozaghian Bäckman[2], Lina Nilsson[1], Mattias Holmlund[3], Nils Skoglund[2], Torgny Näsholm[3,4] & Regina Gratz[3,4]*

[1]Umeå Plant Science Centre (UPSC), Forest Genetics and Plant Physiology, Swedish University of Agricultural Sciences, Umeå, Sweden

[2]Thermochemical Energy Conversion Laboratory, Department of Applied Physics and Electronics, Umeå University, Sweden

[3]Arevo AB, Dåva Energiväg 8, Umeå, Sweden

[4]Forest Ecology and Management, Swedish University of Agricultural Sciences, Umeå, Sweden

*Corresponding author: Regina Gratz (regina.gratz@slu.se)

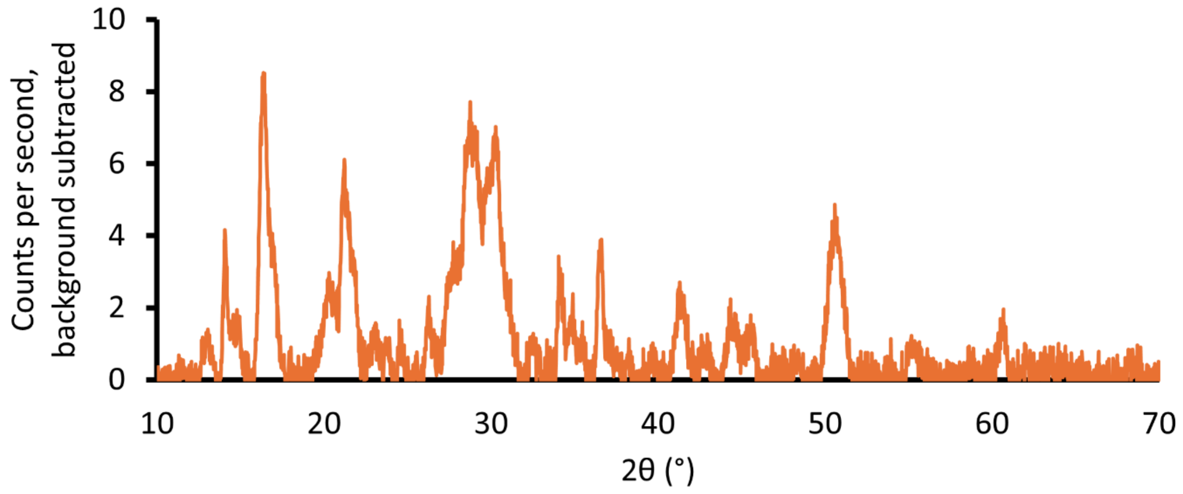


**Supplementary Fig. S1:** X-ray diffractogram of Arginine Fe-HMP with background subtracted.

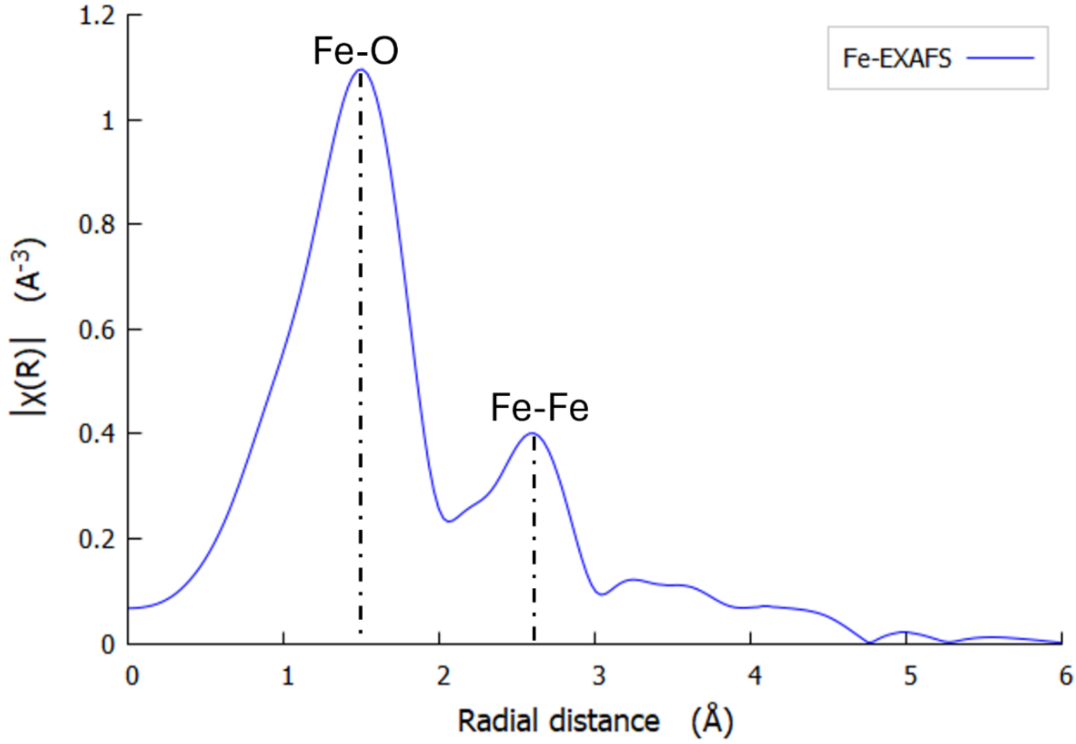


**Supplementary Fig. S2:** Iron K-edge EXAFS for Arginine Fe-HMP with main contributing bonds indicated.

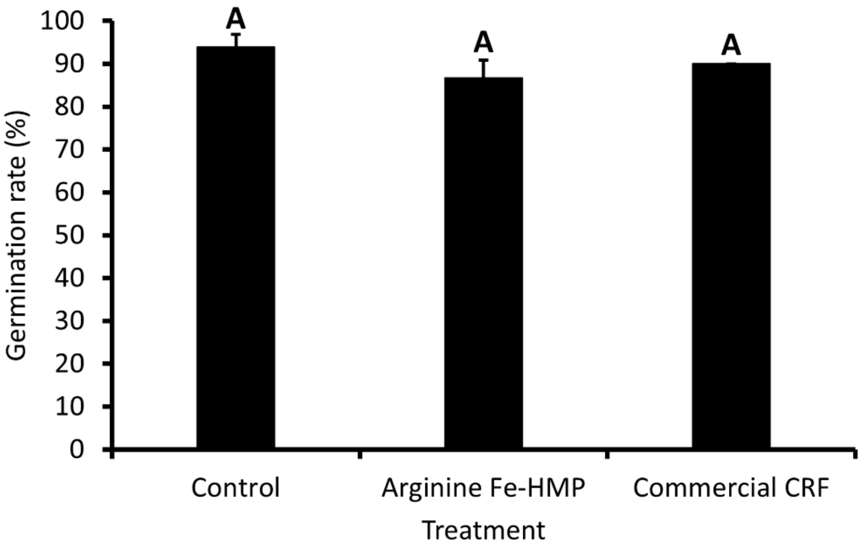


**Supplementary Fig. S3:** Pine germination rate measured on cassettes for the three treatments: control, Arginine Fe-HMP and commercial CRF (mean+SE, n=3). Different letters indicate significant differences based on ANOVA and Tukey HSD test.

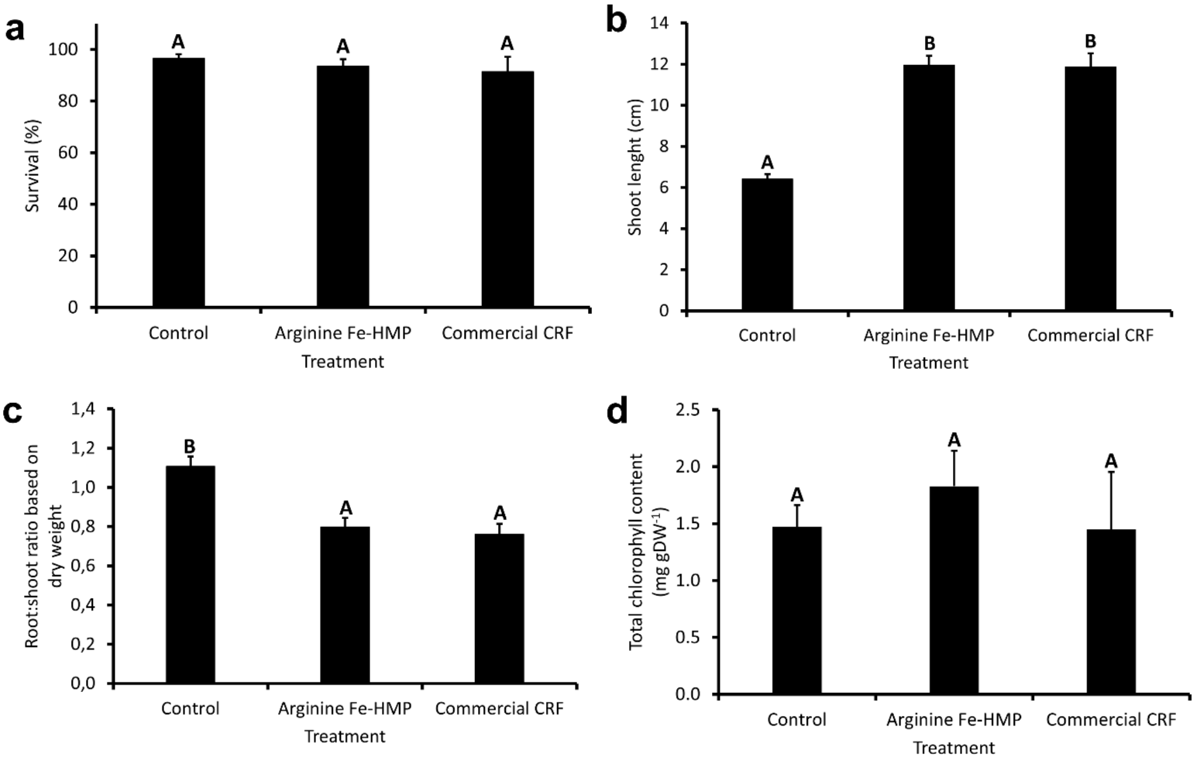


**Supplementary Fig. S4:** Measured traits on pine seedlings after six months of growth: a) survival rates per cassette (mean+SE, n=3), b) seedling shoot length (mean+SE, n=30), c) seedling root-to-shoot ratio based on dry weight (mean+SE, n=30) and d) needle chlorophyll content (mean+SE, n=12) for control, Arginine Fe-HMP and commercial CRF treatment. Different letters indicate significant differences based on ANOVA and Tukey HSD test.

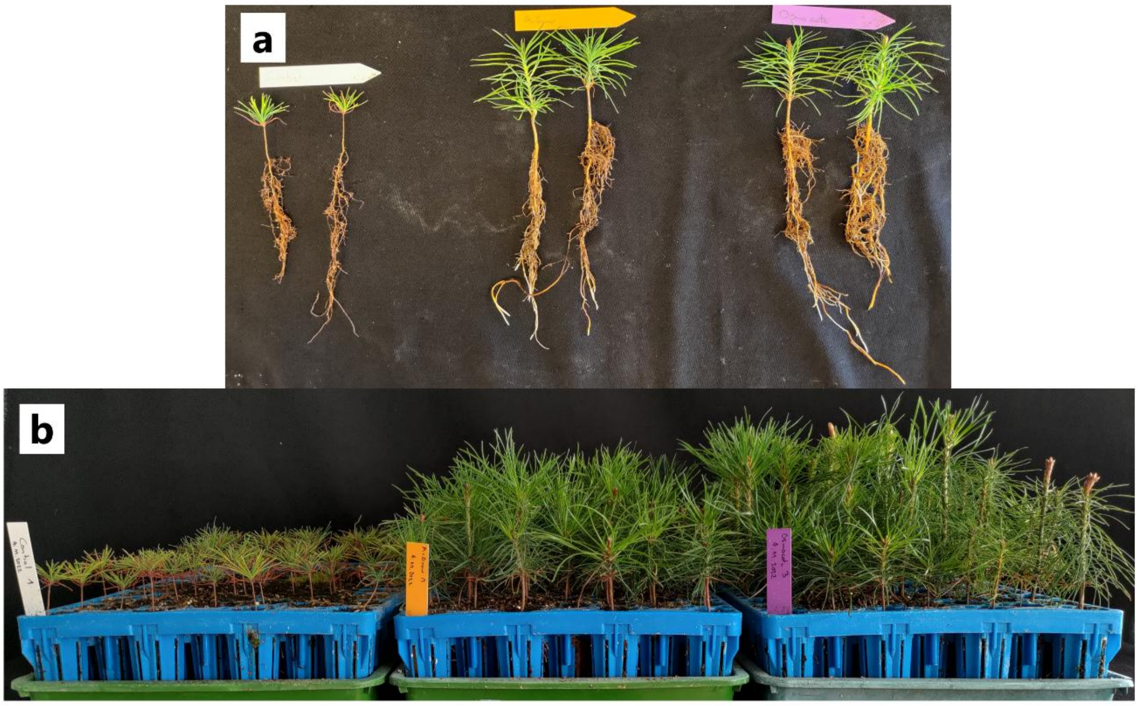


**Supplementary Fig. S5:** Picture of pine seedlings after a) five months of growth and b) six months of growth. From left to right: control, Arginine Fe-HMP and commercial CRF treated seedlings.

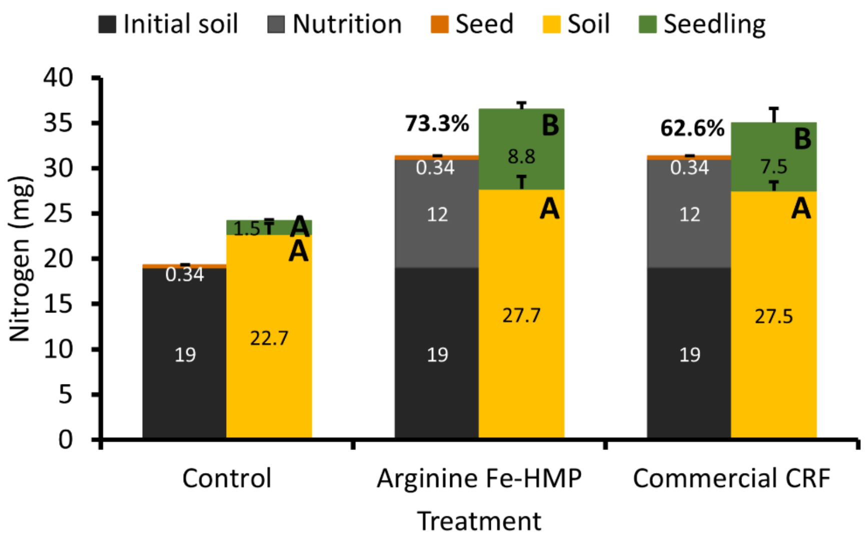


**Supplementary Fig. S6:** Detailed nitrogen budget with expected (left column) and measured (right column) nitrogen per seedling for each treatment. For expected nitrogen, the initial soil nitrogen is the nitrogen present in the soil before planting, nutrition nitrogen is the addition of the nitrogen added through nutrition and seed N is the amount of nitrogen present in the pine seed (mean + SE, n = 3). The measured total nitrogen is the combination of the nitrogen stored in the seedling (mean+SE, n=9) and in the soil (mean+SE, n=3). The number inside bars shows the amount of nitrogen specific for that component, the percentage number by Arginine Fe-HMP and Commercial CRF bars is the nutrition use efficiency for the seedling and the different letters by error bars indicate significant differences based on ANOVA and Tukey HSD test for either soil or seedling.

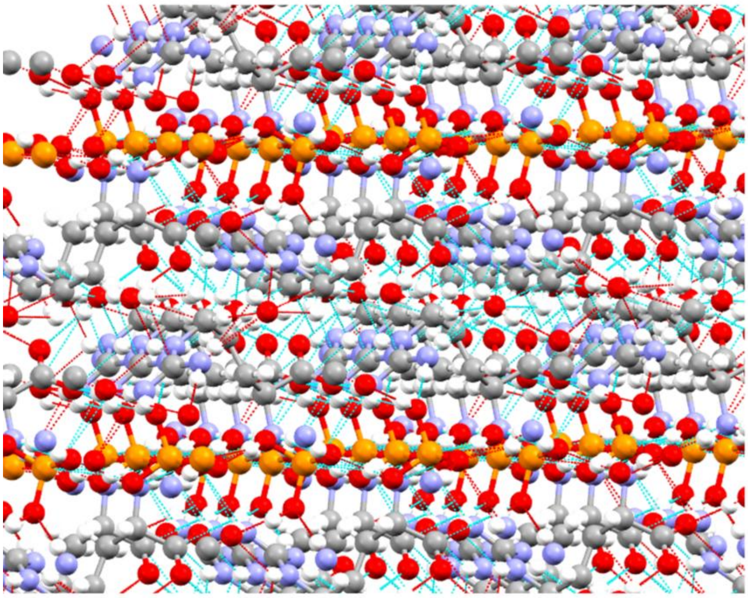


**Supplementary Fig. S7:** Double-stacking of arginine molecules with interstitial single layers of inorganic ions according to Aoki et al. (1971) and Saenger and Wagner (1972) for L-arginine phosphate monohydrate. A possible structure for the Arginine Fe-HMP complex could be similar double arginine layers with a more disordered composition of the interstitial inorganic layer many comprising phosphate and hydrated Fe ions.
